# Latent profile analysis of dyadic resilience in patients with non–small cell lung cancer and their caregivers and implications for intervention strategies: A cross-sectional study

**DOI:** 10.1097/MD.0000000000049936

**Published:** 2026-07-31

**Authors:** Jie Chen, Dabin Yang, Qiang Ren, Kai Wu, Xing Meng, Tian Tian

**Affiliations:** a Cardiothoracic Surgery, Huainan Miner General Hospital, Huaibei City, Anhui Province, China.

**Keywords:** dyad, family caregivers, influencing factors, latent profile analysis, non–small cell lung cancer, psychological resilience

## Abstract

This study investigates the latent classification of resilience levels of non–small cell lung cancer (NSCLC) patient and primary caregiver dyads and analyzes differences in characteristics among and factors associated with inclusion in different subgroups. In this cross-sectional study, from June to December 2024, a convenience sample of 315 dyads of NSCLC patients and their primary family caregivers were recruited from a tertiary general hospital in Anhui Province. Data were collected using a general information questionnaire, the Chinese version of the Connor–Davidson Resilience Scale, and the Social Support Rating Scale. Latent profile analysis was performed using patient and caregiver resilience total scores as observed indicators to identify potential categories of dyadic psychological resilience, and multinomial logistic regression analysis was performed to explore the factors associated with inclusion in each category. The psychological resilience of NSCLC patient–caregiver dyads could be classified into 4 latent categories: category 1, “Dyadic Low Resilience-Low Support Group” (16.51%); category 2, “Dyadic Moderate Resilience-Low Perceived Support Group” (48.89%); category 3, “Dyadic Moderate Resilience-Good Support Utilization Group” (22.86%); and category 4, “Dyadic High Resilience Group” (11.74%). The results of the multinomial logistic regression, with the “Dyadic High Resilience Group” as the reference, showed that older patient age (odds ratio [OR] = 1.08; 95% confidence interval: 1.03–1.14 for class 1 vs class 4), advanced disease stage (III–IV; OR = 2.85–4.12; *P* < .05), lower caregiver education level (OR = 2.78–4.56; *P* < .05), lower total dyad social support score (OR = 0.86–0.91; *P* < .001), and longer daily direct caregiving time (>8 hours; OR = 2.11–3.22; *P* < .05) were significant factors associated with belonging to lower-resilience categories (all *P* < .05). Significant heterogeneity in psychological resilience was observed within dyads composed of NSCLC patients and their caregivers. In clinical practice, an isolationist perspective may benefit from shifting toward a holistic “patient-caregiver” dyadic framework for assessment and intervention. Implementing precise, stratified psychosocial support strategies tailored to the core characteristics of different latent profile groups may be valuable for improving dyadic coping efficacy and shared quality of life.

## 1. Introduction

Non–small cell lung cancer (NSCLC) is among the most common lung malignancies worldwide owing to its high global incidence and mortality. Moreover, its uncertain prognosis^[1,2]^ is a source of persistent and multidimensional stress on patients and their families throughout its lengthy treatment course. During treatment, the patient and their primary family caregiver form a close connection as a dyad, sharing emotions and jointly enduring the impact of the disease. Their psychological adaptation states influence each other, creating a dynamic, synergistic coping system.^[3,4]^ Therefore, assessing them as a “dyadic unit” better reflects the real context of disease coping than assessing the individuals in the dyad separately. Psychological resilience, defined as the ability of an individual or system to adapt effectively, maintain, or restore good functioning when facing significant adversity, is considered a key factor in alleviating cancer-related distress and promoting positive adaptation.^[5]^ However, most existing studies are based on a variable-centered perspective and thus explore the correlation between resilience and related factors, overlooking the potential existence of heterogeneous subgroups with different resilience characteristics within the patient–caregiver dyad. Latent profile analysis (LPA) is a person-centered research method that can identify latent subgroups with different intrinsic characteristics on the basis of multiple observed indicators; such an analysis can provide an in-depth understanding of group heterogeneity.^[6]^ It is important to clarify the conceptual distinction between individual resilience, dyadic resilience, and social support. Individual resilience refers to a person’s capacity to cope with adversity; dyadic resilience, by contrast, emphasizes the dynamic, synergistic adaptation process exhibited by the patient–caregiver dyad as a unit when facing shared stressors, encompassing not only the sum of individual resilience but also the interaction and resource integration between the two. Social support, as a critical external resource, serves as a key buffer in the formation and maintenance of dyadic resilience. Although conceptually distinct, these 3 elements coexist and interact within the dyadic system. Therefore, this study aims to explore the latent profile characteristics of psychological resilience within NSCLC patient–caregiver dyads via LPA and further analyze key factors associated with category membership, with the goal of providing empirical evidence for the development of targeted and efficient dyadic collaborative psychological intervention models.^[7–9]^

## 2. Subjects and methods

### 2.1. Subjects

A cross-sectional study design was used. From June to December 2024, a convenience sample of NSCLC patients who were hospitalized in the departments of respiratory medicine, medical oncology, and thoracic surgery at a tertiary general hospital in Anhui Province and their primary family caregivers were selected as survey participants. This study was approved by the Ethics Committee of Huaibei Miner General Hospital (Approval No.: KYLL2024012). The patient inclusion criteria were as follows: diagnosis with NSCLC by pathological examination; age ≥ 18 years; clarity of consciousness, basic literacy or verbal communication skills, and the ability to understand the content of the questionnaire; and provision of informed consent and voluntary participation in the study. The patient exclusion criteria were as follows: severe cognitive impairment or a history of mental illness, participation in other interventional research that may affect psychological status, and critical illness and an estimated survival of < 3 months (as judged by the attending physician). The caregiver inclusion criteria were as follows: familial relationship (e.g., spouse, child, and sibling) and provision of primary care for the patient, age ≥ 18 years, care provided for >2 hours total each day for at least 1 month, the absence of serious physical conditions or mental illnesses, and provision of informed consent. The caregiver exclusion criteria were as follows: a professional nursing degree and payment for care services or nonfamilial relationship and the provision of care for multiple patients. The sample size, estimated on the basis of the number of observed variables and methodological recommendations for LPA, was at least 30 to 50 samples per latent category. In anticipation of 4 to 5 categories and considering a 20% rate of invalid questionnaires, the final sample size was determined to be 315 dyads. All participants provided written informed consent.

### 2.2. Instruments

#### 2.2.1. General information questionnaire

The questionnaire was developed by the researchers and divided into a patient section and a caregiver section with the following items: Patient section: age, sex, education level, marital status, NSCLC pathological type, tumor-node-metastasis (TNM) stage (based on the American Joint Committee on Cancer criteria, 8th edition), and current treatment plan. Caregiver section: age, sex, education level, relationship to the patient, employment status, monthly household income per capita, daily direct caregiving hours, and total duration of caregiving.

#### 2.2.2. Connor–Davidson Resilience Scale

The Connor–Davidson Resilience Scale (CD-RISC) was adapted into a Chinese version by Yu and Zhang.^[10]^ Although the original scale contains 25 items covering 3 dimensions (tenacity, optimism, and self-reliance), this study used the validated 10-item simplified version (CD-RISC-10) to reduce respondent burden. The CD-RISC-10 uses a 5-point Likert scale (0 = “never” to 4 = “almost always”), yielding a total score ranging from 0 to 40, with higher scores indicating higher levels of psychological resilience. The Cronbach’s α coefficient for this scale was 0.93.

#### 2.2.3. Social Support Rating Scale

The Social Support Rating Scale was developed by Shuiyuan^[11]^ and consists of 10 items covering 3 dimensions: objective support (3 items), subjective support (4 items), and the utilization of social support (3 items). A higher total score indicates a higher level of perceived and available social support. In this study, the Cronbach’s α coefficient for this scale was 0.868.

### 2.3. Data collection and quality control

Uniformly trained research assistants administered the questionnaire surveys face-to-face in quiet areas of the wards. Prior to the survey, the purpose, importance, confidentiality principles, and voluntary nature of the study were explained in detail to the patients and their caregivers. The questionnaires were distributed, completed, and collected on-site; in the event of omissions, the participants were politely prompted to fill in the missing fields. Data were entered independently by 2 individuals using EpiData 3.1 software (EpiData Association), with logical error checking to ensure accuracy.

### 2.4. Statistical analysis

SPSS 25.0 software (IBM Corp.) was used for all statistical analyses, including descriptive statistics and univariable analyses (analysis of variance or rank-sum tests were used for continuous data, and the chi-square test or Fisher exact test was used for categorical data). LPA was conducted using Mplus 8.3 software (Muthén & Muthén, https://www.statmodel.com). The Akaike Information Criterion, Bayesian Information Criterion, and adjusted Bayesian Information Criterion were used for judging the goodness of fit of the LPA models, with smaller values indicating a better fit. Entropy values ranged from 0 to 1, with values closer to 1 indicating higher classification accuracy. The Lo–Mendell–Rubin adjusted likelihood ratio test and the bootstrap likelihood ratio test were also performed, with *P* values < 0.05 indicating that the k-class model was significantly better than the k-1 class model. After the optimal number of classes was determined, with the smallest group as the reference group, variables with statistical significance in the univariable analysis were included as independent variables in a multinomial logistic regression analysis to explore the factors associated with category membership. Variables such as patient sex and medical payment method were also adjusted for in the model. The significance level α was set at 0.05.

## 3. Results

### 3.1. General characteristics of the survey participants

A total of 330 questionnaires were distributed to the dyads, and 315 valid questionnaires were returned, yielding an effective response rate of 95.45%. The mean age of the patients was 62.4 ± 9.8 years, with males accounting for 58.1% (n = 183). The mean age of the caregivers was 51.3 ± 11.2 years, with most being female (67.0%, n = 211) and the patient’s spouse (52.7%; n = 166). Most patients had TNM stage III or IV disease (79.4% combined). Other detailed information is shown in Table 1.

**Table 1:** General characteristics of the NSCLC patients and their caregivers (n = 315).

Item	Patient	%	Caregiver	%
Age (yr, *x* ± *s*)	62.4 ± 9.8	–	51.3 ± 11.2	–
Gender				
Male	183	58.1	104	33.0
Female	132	41.9	211	67.0
Education level				
Junior high school or below	187	59.4	142	45.1
High school technical school	89	28.3	108	34.3
College or above	39	12.4	65	20.6
TNM stage (patient)				
I–II	65	20.6	–	–
III	128	40.6	–	–
IV	122	38.7	–	–
Relationship to the patient				
Spouse	–	–	166	52.7
Child	–	–	112	35.6
Other relative	–	–	37	11.7
Daily caregiving hours				
≤4 h	–	–	103	32.7
>4 and ≤ 8 h	–	–	134	42.5
>8 h	–	–	78	24.8
Monthly household income per capita (CNY)				
<5000	–	–	158	50.2
5000–10,000	–	–	124	39.4
>10,000	–	–	33	10.5

CNY = Chinese Yuan, NSCLC = non–small cell lung cancer, TNM = tumor-node-metastasis.

### 3.2. Results of the latent profile analysis for dyadic psychological resilience

Using the total psychological resilience scores (CD-RISC-10 total scores) of both the patients and their caregivers as observed indicators, models with 1 to 5 latent classes were fitted sequentially. Model fit indices are presented in Table 2. As the number of classes increased, the Akaike Information Criterion, Bayesian Information Criterion, and adjusted Bayesian Information Criterion values continued to decrease. Although the 5-class model had lower fit indices, the smallest class proportion was only 5.1%, indicating an insufficient sample size that might lead to model instability. Furthermore, the entropy value was relatively high (0.912) for the 4-class model, and the Lo–Mendell–Rubin adjusted likelihood ratio test was significant (*P* = .003). Considering model parsimony, class interpretability, and statistical indices comprehensively, the 4-class model was determined to be the optimal solution.

**Table 2:** Model fit indices for the latent profile analysis of dyadic psychological resilience (n = 315).

Model	AIC	BIC	aBIC	Entropy	LMRT *P*-value	BLRT *P*-value	Smallest class, %
1	5210.36	5231.58	5215.47	–	–	–	100
2	4987.12	5019.45	4994.21	0.873	<.001	<.001	38.4
3	4876.89	4920.33	4885.97	0.898	.012	<.001	21.3
4	**4795.24**	**4849.79**	**4806.31**	**0.912**	**.003**	**<.001**	**16.5**
5	4752.11	4817.77	4765.17	0.920	.128	<.001	5.1

The 4-class model was selected as optimal due to class interpretability, sufficient class size, and significant LMRT value.

The bold values highlight the optimal model (the 4-class solution), which has the smallest AIC, BIC, and aBIC, and a significant LMRT *P*-value (<.05), indicating that the 4-class model fits significantly better than the 3-class model. The entropy value (>0.9) also supports high classification accuracy. These bolded values collectively denote the selected best-fitting model.

aBIC = adjusted Bayesian Information Criterion, AIC = Akaike Information Criterion, BIC = Bayesian Information Criterion, BLRT = bootstrap likelihood ratio test, LMRT = Lo–Mendell–Rubin adjusted likelihood ratio test.

Detailed data for the 4 latent classes are shown in Figure 1. In class 1, the “Dyadic Low Resilience-Low Support Group” (n = 52; 16.51%), the psychological resilience scores for the patients and their caregivers were the lowest among the groups, while the total social support score was significantly lower than that of the other groups. In class 2, the “Dyadic Moderate Resilience-Low Perceived Support Group” (n = 154; 48.89%), the psychological resilience scores for the patients and their caregivers were moderate, but the subjective support dimension score was low. In class 3, the “Dyadic Moderate Resilience-Good Support Utilization Group” (n = 72; 22.86%), the psychological resilience scores for the patients and their caregivers were similar to those in class 2, but the score for social support utilization was the highest among the groups. In class 4, the “Dyadic High Resilience Group” (n = 37; 11.74%), the psychological resilience scores for the patients and their caregivers were significantly higher than those in the other 3 groups, and the scores across all social support dimensions were also high.

**Figure:**
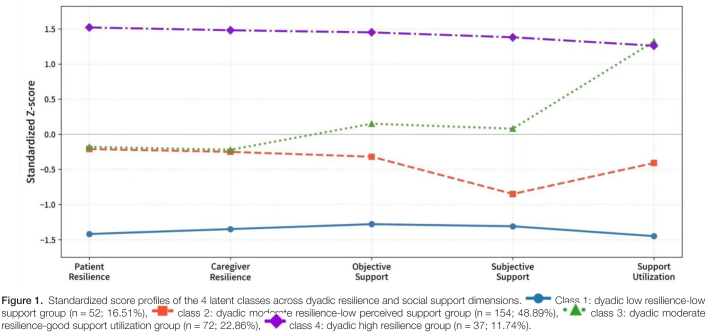


### 3.3. Comparison of general characteristics among different latent profile dyads

Univariable analysis revealed statistically significant differences among the different latent profiles in terms of patient age, TNM stage, caregiver age, education level, relationship to patient, daily caregiving hours, monthly household income per capita, and total social support scores for both patients and caregivers (all *P* < .05). No significant differences were found in terms of sex, marital status, or medical payment method.

### 3.4. Multinomial logistic regression analysis of factors influencing latent profiles of dyadic resilience

Using class membership as the dependent variable (with the “Dyadic High Resilience Group” as the reference), variables that were significant in the univariable analysis were entered into a multinomial logistic regression model. The results (see Table 3) revealed that older patient age, advanced disease stage (III–IV), lower caregiver education level, lower total dyadic social support score, and daily caregiving time exceeding 8 hours were risk factors for the dyad belonging to the lower-resilience category (especially class 1 and class 2).

**Table 3:** Multinomial logistic regression analysis of factors associated with latent profiles of dyadic resilience (with the “High Resilience Group” as the reference).

Influencing factor	Class 1 vs. class 4		Class 2 vs. class 4		Class 3 vs. class 4	
	OR (95% CI)	*P*-value	OR (95% CI)	*P*-value	OR (95% CI)	*P*-value
Patient age (per 1-yr increase)	1.08 (1.03–1.14)	.002	1.05 (1.01–1.09)	.018	1.03 (0.98–1.08)	.212
Patient TNM stage (ref: I–II)						
III	2.85 (1.24–6.55)	.014	2.01 (1.05–3.84)	.003	1.67 (0.85–3.28)	.136
IV	4.12 (1.72–9.86)	.001	2.78 (1.41–5.49)	.003	2.12 (1.04–4.32)	.038
Caregiver education (ref: college or above)						
Junior high or below	4.56 (1.82–11.45)	.001	2.34 (1.18–4.65)	.015	1.89 (0.92–3.88)	.083
High school/technical school	2.78 (1.15–6.72)	.023	1.89 (1.01–3.53)	.047	1.45 (0.74–2.84)	.278
Dyadic total social support score (per 1-point increase)						
Daily caregiving hours (ref: ≤4 h)	0.86 (0.80–0.92)	<.001	0.91 (0.86–0.96)	<.001	0.94 (0.89–0.99)	.023
>4 and ≤ 8 h	1.67 (0.75–3.72)	.211	1.45 (0.82–2.56)	.198	1.23 (0.67–2.26)	.504
>8 h	3.22 (1.38–7.52)	.007	2.11 (1.12–3.98)	.021	1.78 (0.89–3.56)	.102

The model was adjusted for patient sex and medical payment method. Class 1: Dyadic Low Resilience-Low Support Group; Class 2: Dyadic Moderate Resilience-Low Perceived Support Group; Class 3: Dyadic Moderate Resilience-Good Support Utilization Group; Class 4: Dyadic High Resilience Group.

CI = confidence interval, OR = odds ratio, TNM = tumor-node-metastasis.

## 4. Discussion

### 4.1. Heterogeneous classification of dyadic psychological resilience in NSCLC patient–caregiver dyads

This study is the first to include an LPA in a Chinese population with NSCLC and reveal that dyadic psychological resilience among patient–caregiver pairs exists in 4 distinct latent classes. These findings go beyond a simple “high” or “low” dichotomy for dyadic resilience, highlighting the complexity and diversity of its internal structure. Among them, the “Dyadic Moderate Resilience-Low Perceived Support Group” represented the highest proportion (48.89%), suggesting that nearly half of the dyads possess some degree of intrinsic resilience but subjectively feel that they have insufficient understanding, care, and respect from family, friends, or society. This may be because of disease-related stigma, shrinking social circles, or poor communication. Conversely, the smallest group, the “Dyadic High Resilience Group” (11.74%), represents the ideal state, with characteristics that align with the “Hardiness Personality” theory and the “Family Resilience” model – both of which are exemplary and necessary for clinical support and health promotion.^[12]^

### 4.2. Analysis of key factors influencing dyadic resilience category membership

The results of the regression analysis clarify the core variables associated with dyadic resilience classification. First, disease severity (TNM stage) was a prominent factor. Advanced cancer, with its associated symptom burden, complex treatment, and uncertain prognosis, continuously depletes the psychological resources of patients and their caregivers, making it more difficult for them to maintain a high-resilience state. Second, caregiver characteristics play a crucial role. A high education level among caregivers is typically associated with a greater capacity to acquire information, solve problems, and link resources, enabling more effective collaboration with the patient to conquer challenges. Conversely, daily caregiving time exceeding 8 hours was significantly associated with a heavy burden and low resilience, suggesting that prolonged, high-intensity caregiving responsibilities may lead to physical and mental exhaustion, thereby weakening the adaptive capacity of the entire dyadic system. Third, social support, a core indicator of external resources, was the most influential factor distinguishing categories, particularly the low-resilience group. This finding supports the “stress-buffering” model of social support, wherein a strong support network can provide emotional comfort, instrumental assistance, and information directly, while indirectly enhancing the sense of control and meaning for the individuals and the dyad as a whole.^[13]^ Finally, in terms of patient age, the results indicate that younger patients may have advantages in terms of psychological flexibility, future orientation, and technology use, potentially forming a more dynamic coping alliance with their caregivers.

### 4.3. Construction of a precise psychosocial support system based on dyadic classification

On the basis of the above classifications and associated factors, clinical interventions may benefit from shifting from “individual” to “dyadic” and from “universal” to “precise.” The following are hypothesis-generating clinical implications that require validation through future intervention studies.

#### 4.3.1. Implementation of a dyadic integrated assessment and classification management

Upon patient admission or during regular follow-ups, the psychological resilience (e.g., using the CD-RISC-10 short version) and social support levels of both the patient and their primary caregiver should be assessed simultaneously. Rapid assessment tools could be used, and key questions (e.g., “How do you both view the current difficulties?” “Do you feel understood and supported?” “How many hours are spent on caregiving daily?”) could be asked to preliminarily identify the likely resilience category of the dyad, and the findings may be incorporated into the clinical intervention plan.

#### 4.3.2. Implementation of stratified and targeted interventions

For the “Dyadic Low Resilience-Low Support Group,” intensive, multilevel crisis intervention could be considered, including immediate emotional and psychoeducation; identification of resources such as community services, financial assistance, and nurse- or social worker-led respite care; and family involvement initiatives to improve family communication and support.

The “Dyadic Moderate Resilience-Low Perceived Support Group” may benefit from interventions focused on enhancing the sense of subjective support, such as couples or family communication workshops that teach the skills necessary for expressing needs and emotions, patient support groups to better understand their peers and to share experiences,^[14]^ and patient–provider communication to receive empathy and affirmation.

For the “Dyadic Moderate Resilience-Good Support Utilization Group,” interventions focused on consolidating and optimizing existing resource utilization patterns could be offered. Such interventions may include efforts that affirm their effective coping strategies, provide advanced disease management knowledge and self-care skills training, and help with the transition from “good coping” to “posttraumatic growth.”

The “Dyadic High Resilience Group” could be invited to become “peer supporters” or “recovery role models” to further realize their self-worth through helping others. In addition, information on long-term adjustment and relapse prevention should be provided to maintain their positive state.

#### 4.3.3. Modulation of key associated factors

For patients with advanced disease and their caregivers, early and regular psychological distress screening should be conducted, integrating palliative support concepts to help them address issues such as the meaning of life and advance care planning.^[15]^ For caregivers, especially those with lower education levels and long caregiving hours, skills training, time management guidance, and psychological courses should be provided. Moreover, a “family-community-hospital” respite service system could be considered to alleviate the caregiving burden.^[16]^ The use of hospital–community referral channels and online support platforms can not only improve the social network for dyads but can also encourage family engagement and participation in social activities.

## 5. Conclusion

The results of this study confirm that the psychological resilience status of patients with NSCLC and their primary family caregivers is significantly heterogeneous and can be systematically classified into 4 latent categories. This classification is significantly associated with multiple factors, including patient disease stage, age, caregiver education level, caregiving burden, and dyadic social support level. This classification system provides a potential assessment framework and direction for clinical intervention. In clinical practice, practitioners may consider adopting a holistic perspective that identifies dyads with different resilience characteristics through precise assessment and designs stratified, individualized psychosocial support strategies accordingly. This approach may effectively empower the patient–caregiver dyadic system, jointly enhancing their adaptive capacity and overall quality of life while facing the challenges of cancer. However, the findings should be interpreted in light of the study’s limitations. First, the cross-sectional design cannot infer causality or the dynamic evolution of resilience categories. Second, the sample was from a single hospital, which may limit the generalizability of the findings. Third, the proposed classification system requires external validation before it can be considered ready for clinical implementation. Future research could include a longitudinal latent transition analysis to track the dynamic evolution of dyadic resilience subgroups, and randomized controlled trials based on this classification should be performed to verify intervention effectiveness.

## Acknowledgments

The authors thank all the investigators and participants of the Huaibei Miner General Hospital.

## Author contributions

**Conceptualization:** Jie Chen.

**Investigation:** Jie Chen, Kai Wu, Xing Meng.

**Supervision:** Jie Chen.

**Writing – review & editing:** Jie Chen.

**Data curation:** Dabin Yang, Qiang Ren.

**Formal analysis:** Tian Tian.
